# Anti-VGCC negative and Anti-SOX1 positive Lambert-Eaton myasthenic syndrome as the initial presentation of small cell lung cancer: a case report

**DOI:** 10.3389/fonc.2026.1825530

**Published:** 2026-06-17

**Authors:** Yezhou Chen, Jian Zhang, Qinglun Hou, Hen Li, Jie Liu, Wenhui Shi, Meili Sun

**Affiliations:** 1 Department of Graduate, Shandong First Medical University & Shandong Academy of Medical Sciences, Jinan, Shandong, China; 2 Department of Oncology, Central Hospital Affiliated to Shandong First Medical University, Jinan, Shandong, China; 3 Department of Pathology, Pingyin County People’s Hospital, Jinan, Shandong, China; 4 Department of Neurology, Central Hospital Affiliated to Shandong First Medical University, Jinan, Shandong, China

**Keywords:** anti-SOX1 antibodies, Anti-VGCC antibodies, DELTA-P score, Lambert-Eaton myasthenic syndrome, small cell lung cancer

## Abstract

Lambert-Eaton myasthenic syndrome (LEMS) is a rare autoimmune disorder characterized by impaired acetylcholine release at the neuromuscular junction, leading to proximal muscle weakness, autonomic dysfunction, and diminished or absent tendon reflexes. Here, we present a rare case of a 73-year-old male smoker with small cell lung cancer (SCLC), whose initial manifestation was LEMS. Notably, the patient tested seronegative for anti-voltage-gated calcium channel (VGCC) antibodies but was seropositive for anti-SOX1 antibodies. The diagnosis of LEMS was supported by characteristic clinical symptoms and electromyography findings. Subsequent imaging (chest CT and PET-CT) identified a left upper lobe lung mass, which was pathologically confirmed as SCLC, clinically staged as T1cN3M0 (IIIB). Following standard antitumor therapy, the patient’s muscle weakness markedly improved, and his overall survival has exceeded 36 months. This case underscores the critical importance of testing for anti-SOX1 antibodies and applying the DELTA-P score for malignancy screening in high-risk patients presenting with LEMS-like symptoms, even when seronegative for anti-VGCC antibodies.

## 1 Introduction

Lambert-Eaton myasthenic syndrome (LEMS) is a rare autoimmune disorder affecting the neuromuscular junction, which can occur as a primary autoimmune condition or as a paraneoplastic syndrome. Clinically, it is characterized by proximal muscle weakness, reduced or absent deep tendon reflexes, and autonomic dysfunction (1). Weakness in the proximal muscles of the lower limbs is the characteristic and often the initial clinical manifestation of LEMS. Typically, muscle weakness progresses from proximal to distal regions, extending from the lower to the upper limbs, and may eventually involve the ocular and bulbar muscles; however, respiratory failure is exceedingly rare. Autonomic dysfunction primarily manifests as dry eyes, dry mouth, constipation, and erectile dysfunction in male patients (2, 3). Approximately 50-60% of LEMS cases are paraneoplastic, with small cell lung cancer (SCLC) being the most prevalent associated malignancy, accounting for approximately 90% of paraneoplastic cases (2). Diagnosis primarily relies on the detection of antibodies against voltage-gated calcium channels (VGCCs) (4).Anti-VGCC antibodies are present in 85%-90% of all patients with LEMS and in nearly 100% of those with SCLC-associated paraneoplastic LEMS (5).

However, the diagnostic process becomes challenging when atypical presentations occur, particularly when anti-VGCC antibody test results are negative. SRY-like high mobility group box 1 (SOX1) is a transcription factor intricately involved in neural development and tumorigenesis. Anti-SOX1 antibodies are characterized by high specificity for SCLC and can be detected at early disease stages. Consequently, they hold significant diagnostic value in tumor-associated LEMS, particularly in patients who are negative for VGCC antibodies (6). Furthermore, neurological symptoms in patients with LEMS often precede the diagnosis of the underlying tumor (7–9). To address this, the Dutch-English LEMS Tumor Association Prediction (DELTA-P) score was developed to assess risk based on independent prognostic factors. Within the first three months of symptom onset, one point is assigned for each of the following criteria: age > 50 years, current smoking status, weight loss > 5%, bulbar involvement, erectile dysfunction, and a Karnofsky performance status (KPS) < 70. A total score of ≥ 3 is highly indicative of concurrent SCLC. By focusing on relevant neurological symptoms, autoantibody profiles, and high-risk factors, regular clinical and radiological screening can be implemented for patients to facilitate early tumor detection and initiate targeted therapy (10, 11).

Here, we report a case of LEMS associated with SCLC. The patient was seronegative for anti-VGCC antibodies but positive for anti-SOX1 antibody. This case highlights the diagnostic challenges in atypical LEMS lacking conventional serological markers and underscores the critical role of anti-SOX1 antibody and the DELTA-P score in guiding SCLC screening. Given the distinct clinical features and multidisciplinary nature of tumor-associated LEMS, this report aims to comprehensively delineate the patient’s clinical characteristics, diagnostic workup, therapeutic strategies, and long-term prognosis. In doing so, we hope to provide valuable insights for oncologists and neuro-oncologists in the future diagnosis and management of this complex condition.

## 2 Case presentation

The patient was a 73-year-old male with no significant past medical history of hypertension, diabetes mellitus, coronary heart disease, renal disease, or cerebral infarction. He had a smoking history of over 20 pack-years (approximately 1 pack per day) and denied any history of alcohol consumption. Furthermore, there was no history of relevant occupational exposure. On February 20, 2023, the patient presented with progressive weakness predominantly affecting the lower limbs, resulting in severe gait disturbance requiring assistance. Additionally, he reported constipation and urinary retention and had experienced unintentional weight loss exceeding 5% over the past month. However, there was an absence of erectile dysfunction, dysphagia, dysarthria, and respiratory or circulatory dysfunction. The patient was unable to perform activities of daily living, with a Karnofsky Performance Status (KPS) score of 30. There was no significant family history of neurological disorders.

Physical examination revealed bilateral muscle weakness (right upper limb muscle strength grade proximal 4, distal 4+; left upper limb muscle strength grade proximal 3, distal 4; right lower limb muscle strength grade proximal 2, distal 2+; left lower limb muscle strength grade proximal 3, distal 4). Deep tendon reflexes (biceps, triceps, brachioradialis, patellar and achilles) were absent in all four limbs; however, superficial abdominal reflexes were preserved. Sensory examination showed a stocking-glove distribution of hypoalgesia in all four limbs. Bilateral pupillary light reflexes were normal, with no ptosis or diplopia.

**Figure 1:**
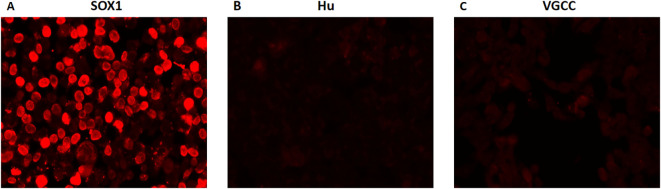
Antibodies associated with paraneoplastic syndromes were detected in the patient using serological testing. **(A)** Anti-SOX1 antibodies were positive (1:100). **(B, C)** Anti-Hu antibodies and anti-voltage-gated calcium channel (VGCC) and were negative.

Hematological examinations revealed no significant abnormalities in routine blood tests, blood electrolytes, liver and kidney function, or conventional tumor markers (AFP, CEA, CA19-9, PSA, NSE, non-small cell lung cancer-related antigens). The patient underwent paraneoplastic syndrome-related antibody testing using a cell-based assay (CBA). Based on the antibody titers, the results were classified as negative (−), low-titer positive (+, 1:10–1:32), moderate-titer positive (++, 1:100–1:320), or high-titer positive (+++, ≥1:1000). In this patient, P/Q-type voltage-gated calcium channel (VGCC) antibodies were negative, while anti-SOX1 antibodies were positive (1:100). Other antibodies, including anti-Hu, anti-Ri, anti-CV2, anti-Ma1, anti-Ma2, anti-Amphiphysin, anti-Tr, anti-Zic4, and anti-GAD65 antibodies, were all negative (**Figure 1**). Furthermore, myasthenia gravis-related antibodies, including acetylcholine receptor (AChR) antibodies, MuSK antibodies, LRP4 antibodies, and RyR antibodies, were all negative.

Electromyography (EMG) showed reduced amplitude of compound muscle action potentials (CMAPs) at rest. Low-frequency repetitive nerve stimulation (RNS) demonstrated a maximum amplitude decrement of 29% in the bilateral trapezius muscles and 22% in the right abductor digiti minimi. Unfortunately, the patient was unable to tolerate high-frequency RNS, and stimulation at frequencies greater than 20 Hz was not performed. However, the patient exhibited postexercise facilitation of knee reflexes, which partially compensated for the lack of high-frequency RNS data. Six months later, when the patient experienced recurrence of muscle weakness symptoms, low-frequency RNS of bilateral orbicularis oculi, trapezius, and right abductor digiti minimi showed significant CMAP amplitude decrement, and a 30% CMAP amplitude increment was observed with 20 Hz repetitive stimulation of the abductor digiti minimi.

**Figure 2:**
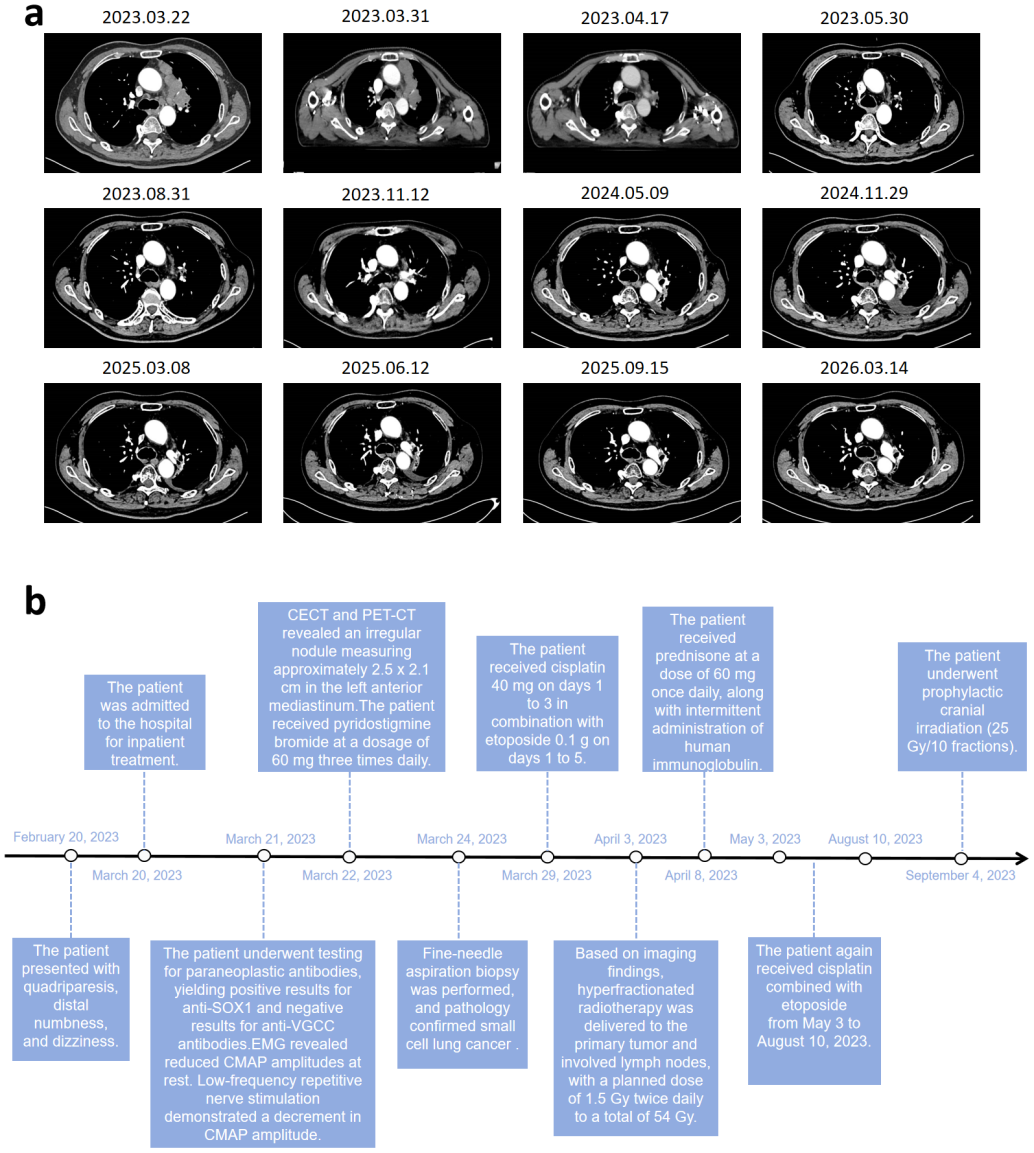
**(A)** CT imaging illustrated the dynamic changes in the size of the primary tumor at the time of SCLC diagnosis, during treatment, and throughout the follow-up period. Current radiological assessment indicates stable disease. **(B)** Timeline of the patient’s clinical course, including symptoms, diagnostic workup, and treatment.

**Figure 3:**
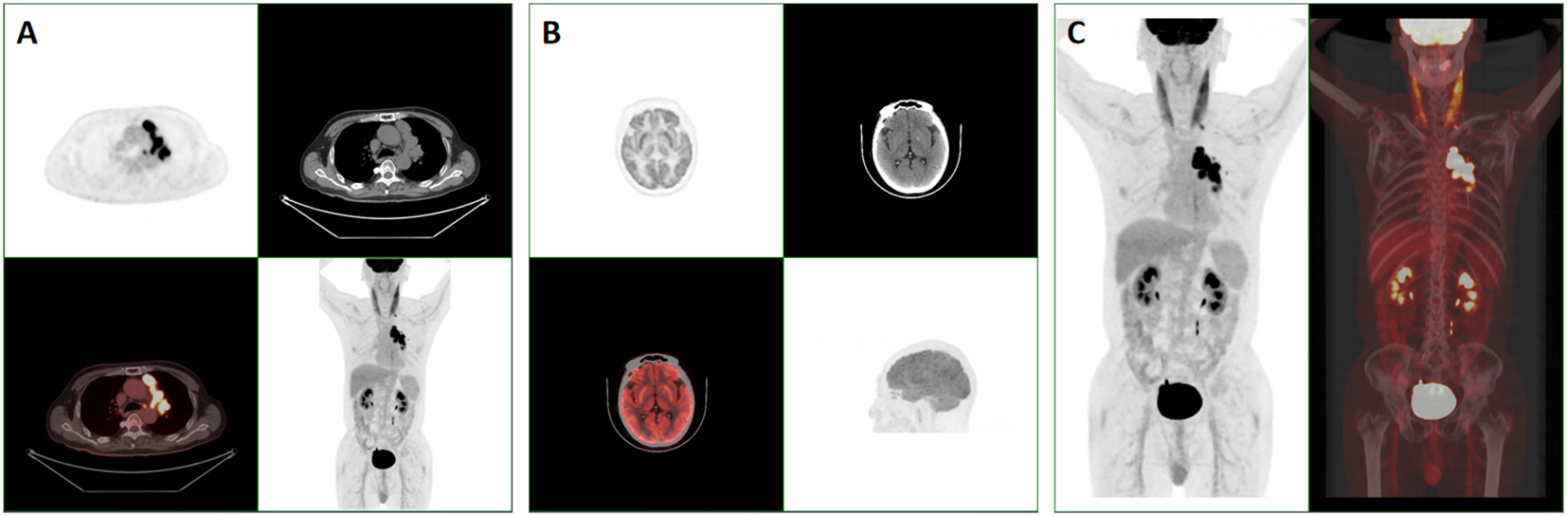
PET-CT was subsequently performed for further evaluation of a lung mass detected on CT. **(A)** A hypermetabolic mass (SUVmax 9.8) is seen in the anterior segment of the left upper lobe near the hilum. **(B)** No significant morphological or metabolic abnormalities are identified in the brain. **(C)** Whole-body imaging shows no evidence of distant metastasis.

Chest computed tomography (CT) demonstrated an irregular nodule (approximately 2.5 × 2.1 cm) in the left upper lobe, characterized by ill-defined interfaces with the pericardium and left hilum, and partial encasement of adjacent vasculature. Furthermore, mediastinal and left hilar lymphadenopathy were observed to encase the left upper lobe pulmonary arteries and veins, leading to vascular wall irregularity and luminal stenosis (**Figure 2A**). Positron emission tomography-computed tomography (PET-CT) confirmed a hypermetabolic lesion in the left upper lung lobe (SUVmax 9.8) and mediastinal and left hilar lymph nodes, highly suggestive of malignancy (**Figure 3**). Cranial magnetic resonance imaging (MRI) plain scan and enhancement showed no obvious abnormalities.

**Figure 4:**
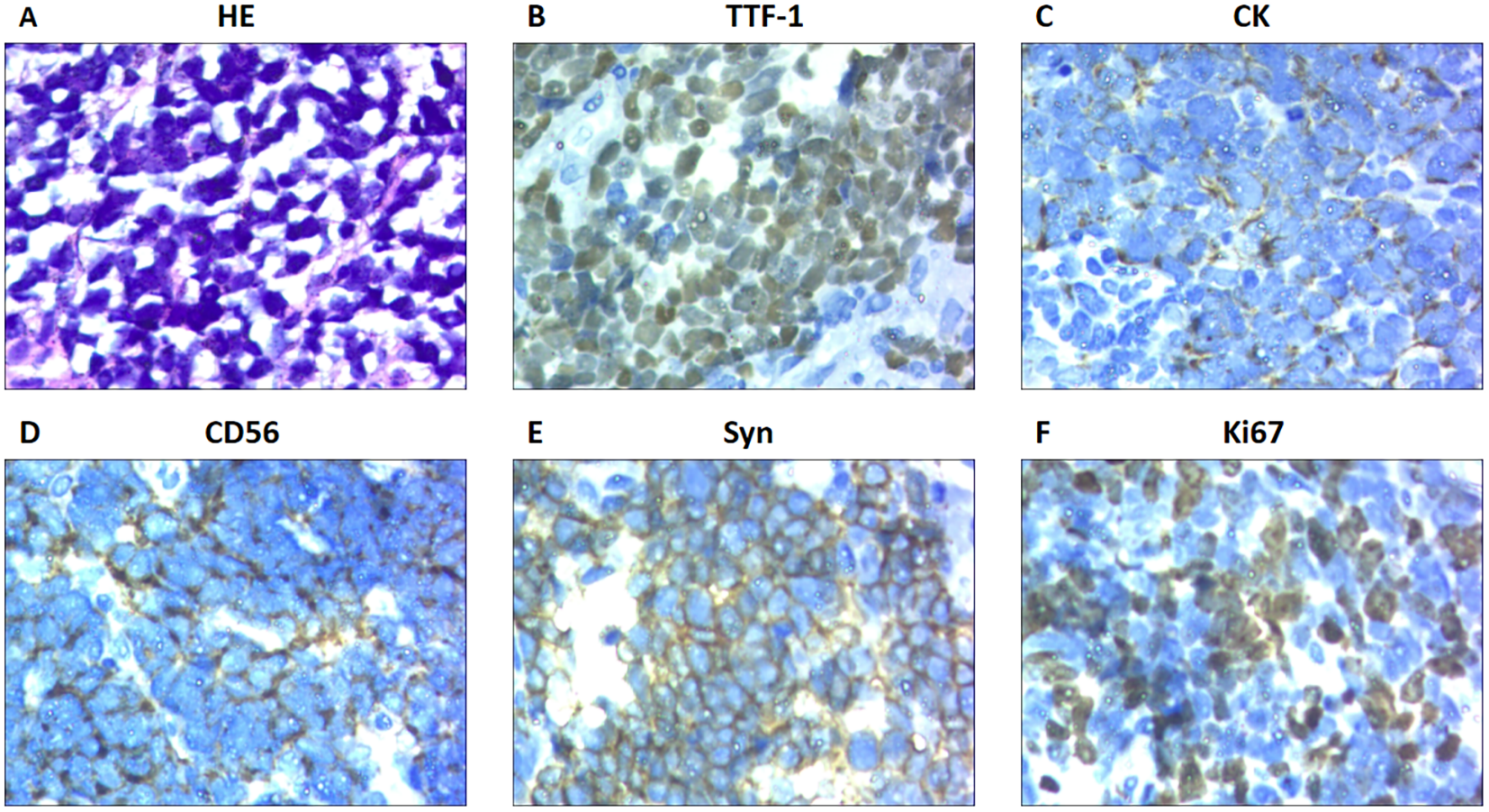
Hematoxylin plus eosin (H&E) and immunohistochemistry (IHC) staining of lung cancer tissue samples(40×). **(A)** Haematoxylin plus eosin (H&E) staining is consistent with small cell lung carcinoma. **(B–E)** Immunohistochemical stain showed that cells were positive for TTF-1,CK,CD56, and Syn. **(F)** The Ki-67 proliferation index is approximately 65%.

Finally, a pathological biopsy confirmed SCLC. Immunohistochemistry showed CK(+), Syn(+), CD56(+), CgA(partial+), TTF-1(+), P53(+, mutant type), Ki-67(+, approximately 65%), CK7 (–), NapsinA (–), CK5/6 (–), P40 (–) (**Figure 4**). Based on the patient’s typical clinical presentation, electrophysiological examination results, anti-SOX1 antibody positivity, and pathological confirmation of SCLC, the patient was diagnosed with LEMS as a paraneoplastic manifestation of SCLC, with a clinical stage of cT1cN3M0, stage IIIB.

On March 29, 2023, the patient received the first cycle of cisplatin (40 mg, days 1–3) combined with etoposide (100 mg, days 1–5). Concurrently, hyperfractionated radiotherapy targeting the primary tumor and involved lymph nodes was initiated on April 3, 2023. The planned regimen consisted of 1.5 Gy twice daily to a total dose of 54 Gy, which was successfully completed on April 26, 2023.The patient experienced grade IV bone marrow suppression with leukopenia after the initial radiotherapy, which resolved rapidly following active treatment with pegylated recombinant human granulocyte colony-stimulating factor (PEG-rhG-CSF). Subsequently the patient underwent five additional cycles of cisplatin and etoposide. Following the completion of systemic chemotherapy, prophylactic cranial irradiation (PCI) was administered starting September 4, 2023, at a dose of 2.5 Gy per fraction for 10 fractions, totaling 25 Gy. Subsequent chemotherapy and PCI were well tolerated, with no significant adverse events observed throughout the entire treatment course. For LEMS symptoms, the patient received intermittent intravenous immunoglobulin (IVIG) and daily oral prednisone (60 mg) starting from April 8, 2023. Since 3,4-diaminopyridine (amifampridine) is not available in our country, pyridostigmine bromide (60 mg three times daily) was administered to alleviate muscle weakness. Following anti-tumor therapy, his muscle weakness improved significantly, with only mild residual lower limb weakness, allowing him to walk independently. The patient completed anti-tumor treatment on September 15, 2023, and underwent regular follow-up. The most recent follow-up on March 14, 2026, demonstrated stable disease on imaging (**Figure 2A**). The patient has achieved an overall survival (OS) of over 36 months since the diagnosis of SCLC and maintains a good quality of life. Currently, the patient is asymptomatic, with no complaints of lower limb weakness, dizziness, or bowel/bladder dysfunction. Based on the assessment of self-care, physical activity, and disease status, the patient has a Karnofsky Performance Status (KPS) of 90, indicating a favorable general condition. The patient’s treatment course is summarized in **Figure 2B**.

## 3 Discussion

LEMS is a rare autoimmune disorder of the neuromuscular junction that typically presents with symmetrical proximal muscle weakness, diminished or absent deep tendon reflexes, and autonomic dysfunction (4, 10, 12). We report a case of atypical paraneoplastic LEMS associated with SCLC that was seronegative for anti-VGCC antibodies but seropositive for anti-SOX1 antibodies.

Retrospective studies indicate a low rate of VGCC antibody negativity in SCLC-associated LEMS: approximately 3% to 7% (13). This serological profile may pose challenges for clinicians relying solely on anti-VGCC antibody testing. This patient was seronegative for anti-VGCC antibodies, which is relatively uncommon in SCLC-related LEMS. Although radioimmunoprecipitation assay (RIPA) is conventionally recommended for the detection of VGCC antibodies in LEMS, we employed a CBA in this case. CBA is increasingly advocated in the literature for the detection of neural autoantibodies, as it offers superior sensitivity and greater safety and convenience by eliminating the need for radioactive isotopes. Negative anti-VGCC antibodies in patients with LEMS may be due to low antibody concentrations, different VGCC epitopes, or the presence of antibodies targeting other proteins (9). A limitation of our study is the inability to re-validate these findings using RIPA. Furthermore, studies have found that in anti-VGCC antibody-negative patients with LEMS, repetitive nerve stimulation also rarely shows the typical electrophysiological findings, including reduced CMAP amplitude, decreased CMAP amplitude with low-frequency RNS, and significantly increased CMAP amplitude with high-frequency RNS (14, 15). In this case, the patient was unable to tolerate high-frequency RNS. Unfortunately, we were unable to perform the post-exercise CMAP facilitation test for further verification. Thus, LEMS cannot be excluded based solely on negative anti-VGCC antibody testing in clinically suspected cases. We recommend proceeding with thorough tumor screening irrespective of serological findings.

In this patient, the detection of anti-SOX1 antibodies underscores the critical need for active tumor screening. SOX1 is a transcription factor closely related to neural development and tumorigenesis. Anti-SOX1 antibodies are highly specific for SCLC and are considered reliable markers for paraneoplastic neurological syndromes (16, 17). In some cases, anti-SOX1 antibodies can be detected in SCLC patients even prior to radiographic evidence. However, there is currently no evidence proving that SOX1 directly causes LEMS. Instead, the presence of anti-SOX1 antibodies indicates that SCLC patients express the SOX1 antigen, which is recognized by the immune system, thereby triggering the production of anti-SOX1 antibodies. The pathogenesis of LEMS is closely linked to SCLC and involves the generation of VGCC autoantibodies that attack the neuromuscular junction, leading to its dysfunction. Therefore, it is hypothesized that the aberrant expression of SOX1 protein in SCLC not only induces anti-SOX1 antibody production but may also involve the expression of molecules structurally similar to neuronal components (such as VGCCs), which subsequently triggers an autoimmune response against these structures (16, 18). Therefore, as a sensitive and specific marker, a positive anti-SOX1 antibody test in patients with LEMS remains a strong indicator of underlying malignancy and should still prompt comprehensive screening for SCLC. This highlights the value of anti-SOX1 antibodies as a complementary screening biomarker for patients presenting with LEMS-like symptoms but negative anti-VGCC results, thereby preventing missed diagnoses of SCLC.

Furthermore, LEMS requires differentiation from other common neurological disorders, with myasthenia gravis (MG) being the primary condition to distinguish. Although both are autoimmune diseases affecting neuromuscular junction transmission, they exhibit distinct differences in clinical manifestations, serological markers, and electrophysiological features. Clinically, LEMS typically presents with the classic triad of proximal symmetric muscle weakness, hyporeflexia or areflexia, and autonomic dysfunction (4). In contrast, MG is characterized by fluctuating, fatigable muscle weakness—often manifesting as ptosis and diplopia, which may progress to involve bulbar and limb muscles—while deep tendon reflexes are usually preserved, and autonomic symptoms are rare. Serologically, anti-VGCC antibodies are detectable in patients with LEMS, whereas MG patients primarily express anti-AChR or anti-MuSK antibodies. Electrophysiological examination serves as another crucial tool for differentiation. LEMS is characterized by a significantly reduced CMAP amplitude at rest; however, following high-frequency RNS or brief maximal voluntary contraction, the CMAP amplitude increases by more than 100% (post-exercise facilitation), a phenomenon attributed to the accumulation of calcium ions in the presynaptic terminal. Conversely, MG demonstrates a progressive decrement in CMAP amplitude during low-frequency RNS, reflecting the limited functional reserve of postsynaptic receptors (19, 20). Additionally, differential diagnoses should include Guillain-Barré syndrome (GBS) and botulism. GBS, an acute inflammatory demyelinating polyneuropathy, is often preceded by an infection and presents with rapidly progressive symmetric flaccid paralysis, sensory abnormalities, and albuminocytologic dissociation in the cerebrospinal fluid, with electrophysiology typically showing slowed nerve conduction velocities or prolonged F-waves without high-frequency facilitation (21). Botulism, caused by the ingestion of botulinum toxin which inhibits presynaptic acetylcholine release, can also lead to symmetric descending paralysis and autonomic dysfunction; however, it is usually associated with a history of ingesting contaminated food and lacks positive autoantibodies, which helps distinguish it despite occasionally showing electrophysiological patterns similar to LEMS. Therefore, the accurate diagnosis of LEMS relies on a comprehensive assessment integrating the clinical triad, antibody profiling, electrophysiological findings, and tumor screening to precisely differentiate it from other neuromuscular disorders.

Notably, the stocking-glove hypoalgesia observed in this patient represents an atypical feature worthy of attention. Although LEMS, as a disorder of the neuromuscular junction, typically spares sensory function, the autoimmune injury within SCLC-associated paraneoplastic syndromes is often multi-targeted. Unfortunately, we were unable to evaluate small fiber lesions using quantitative sensory testing, sudomotor autonomic function testing, and skin biopsy with quantification of intraepidermal nerve fiber density. This hypoalgesia in the distal extremities raises the possibility of a concomitant paraneoplastic peripheral neuropathy. It is worth noting that although routine onconeural antibodies (such as anti-Hu, anti-CV2, and anti-Amphiphysin) were negative in this case, existing literature indicates that anti-SOX1 antibody positivity itself may also be associated with paraneoplastic peripheral neuropathy (22). This further underscores the high complexity of SCLC-mediated paraneoplastic syndromes, which can simultaneously involve both the neuromuscular junction and peripheral nerves, resulting in an overlapping clinical phenotype.

Neurological symptoms in patients with LEMS often precede the appearance of the tumor, presenting as the initial manifestation. In recent years, the DELTA-P score has been used to assess the probability of an underlying tumor in patients with LEMS based on high-risk factors. The scoring system comprises six items, each assigned one point: age > 50 years, smoking history, weight loss > 5% within the past 3 months, erectile dysfunction, bulbar involvement, and a KPS score < 70 (17, 23). In this case, the patient met four criteria: age > 50 years, smoking history, weight loss > 5% within the past 3 months, and a KPS score < 70. The patient had no erectile dysfunction or bulbar involvement. Consequently, the DELTA-P score was 4, which is highly suggestive of SCLC. Based on prospective and retrospective studies, the predicted probabilities of concurrent SCLC are 86% and 93.5%, respectively (23–25). In clinical practice, when faced with atypical patients with LEMS, the DELTA-P score can also be used for initial assessment to guide further screening.

SCLC is a highly aggressive malignancy with generally poor prognosis. However, studies have shown that SCLC patients complicated with paraneoplastic LEMS may have a better prognosis compared to SCLC patients without LEMS. A study of mostly advanced SCLC patients with LEMS reported that the median survival time (MST) could reach 18 months after treatment with platinum-etoposide regimens (26). The patient with limited-stage small cell lung cancer received standard concurrent chemoradiotherapy, followed by PCI after chemotherapy, and his OS has exceeded 36 months, with significant improvement in LEMS symptoms and stable tumor status (27).

The longer survival of SCLC patients complicated with paraneoplastic LEMS may be attributed to several factors. LEMS symptoms are often discovered earlier than primary tumor symptoms, which prompts patients to seek medical attention promptly and undergo active screening, leading to early diagnosis and timely initiation of effective anti-tumor treatment (7–9). The etiology of LEMS lies in the body’s immune response to SCLC tumor cell surface antigens. This immune response targeting tumor antigens, while attacking the neuromuscular junction, also produces an anti-tumor effect on the tumor itself, thereby helping to inhibit tumor growth and improve patient prognosis (4).

In contrast to classic VGCC-positive LEMS cases, this patient presented with seronegative LEMS. Despite the diagnostic challenges posed by the negative VGCC antibodies and the inability to perform high-frequency RNS, a comprehensive assessment integrating the patient’s typical neurological manifestations, positive SOX-1 antibodies, and DELTA-P score prompted us to initiate an aggressive tumor screening. This led to the early diagnosis and multidisciplinary treatment of occult SCLC. Consequently, the patient has achieved a remarkable overall survival exceeding 37 months, with a significant recovery in quality of life (KPS score improved from 30 to 90). This case underscores the importance of incorporating SOX-1 antibody testing and risk stratification tools like the DELTA-P score into the diagnostic workflow for seronegative LEMS, highlighting that timely intervention can lead to exceptional long-term outcomes.

## Data Availability

The raw data supporting the conclusions of this article will be made available by the authors, without undue reservation.
